# Ge pMOSFETs with GeO_*x*_ Passivation Formed by Ozone and Plasma Post Oxidation

**DOI:** 10.1186/s11671-019-2958-2

**Published:** 2019-04-05

**Authors:** Yang Xu, Genquan Han, Huan Liu, Yibo Wang, Yan Liu, Jinping Ao, Yue Hao

**Affiliations:** State Key Discipline Laboratory of Wide Band Gap Semiconductor Technology, School of Microelectronics, Xidian University, Xi’an, 710071 People’s Republic of China

**Keywords:** Germanium, Passivation, Ozone, Plasma, Post oxidation, Metal-oxide-semiconductor field-effect transistor (MOSFET)

## Abstract

A comparison study on electrical performance of Ge pMOSFETs with a GeO_*x*_ passivation layer formed by ozone post oxidation (OPO) and plasma post oxidation (PPO) is performed. PPO and OPO were carried out on an Al_2_O_3_/n-Ge (001) substrate followed by a 5-nm HfO_2_ gate dielectric in situ deposited in an ALD chamber. The quality of the dielectric/Ge interface layer was characterized by X-ray photoelectron spectroscopy and transmission electron microscopy. The PPO treatment leads to a positive threshold voltage (*V*_TH_) shift and a lower *I*_ON_/*I*_OFF_ ratio, implying a poor interface quality. Ge pMOSFETs with OPO exhibit a higher *I*_ON_/*I*_OFF_ ratio (up to four orders of magnitude), improved subthreshold swing, and enhanced carrier mobility characteristics as compared with PPO devices. A thicker Al_2_O_3_ block layer in the OPO process leads to a higher mobility in Ge transistors. By comparing two different oxidation methods, the results show that the OPO is an effective way to increase the interface layer quality which is contributing to the improved effective mobility of Ge pMOSFETs.

## Background

With conventional complementary metal-oxide-semiconductor (CMOS) devices approaching its physical limit, performance enhancement is hard to realize for high-speed semiconductor devices with silicon (Si) as the channel material. Replacing substrate or channel material with other material with high mobility is an imperative option. Germanium (Ge) has been considered as a promising alternative channel material owing to higher carrier mobility than that of Si. The MOSFET usually needs a high-quality oxide/semiconductor interface to reach high effective mobility. However, for quite a long history, Ge MOSFETs suffered from the high interface state density (*D*_it_) caused by the poor thermal stability of GeO_2_ and dangling bonds [1]. Thus, plenty of research has been carried out on Ge interface passivation.

Several approaches to achieving a high-quality Ge/dielectric interface layer have been reported, such as Si passivation by uniformly depositing several Si monolayers on Ge substrate before dielectric epitaxy or self-passivation by forming GeO_2_ on purpose [2, 3]. In order to form a high-quality GeO_2_ layer, there are many oxidation processes to reduce *D*_it_ and improve thermal stability including high-pressure oxidation [4], ozone oxidation [5], H_2_O plasma [6], and electron cyclotron resonance (ECR) plasma post oxidation [7].

In recent years, plenty of works have been reported that high-performance Ge MOSFET can be realized by post oxidation through Al_2_O_3_/Ge interface. In 2014, a Ge CMOS inverter was realized on a Ge-on-insulator (GeOI) substrate with GeO_*x*_ grown by rapid thermal annealing in pure oxygen ambient after 1 nm Al_2_O_3_ was deposited on Ge [8]. In ref. [7], Ge pMOSFETs and nMOSFETs with GeO_*x*_ passivation were fabricated with oxygen plasma post oxidation and temperature dependence of GeO_*x*_ thickness and electrical performance were also discussed. Thermal oxidation of Ge by ozone can be performed at a lower temperature, for ozone is more reactive than oxygen [5]. The impact of temperature on GeO_*x*_ thickness grown by ozone on Ge surface was demonstrated. Ge pMOSFETs with GeO_*x*_ passivation fabricated by ozone post oxidation was also reported [9].

In this work, Ge pMOSFETs with GeO_*x*_ passivation are fabricated using ozone post oxidation (OPO) and oxygen plasma post oxidation (PPO) of the Al_2_O_3_/n-Ge interface. A comparison study on the electrical performance of Ge pMOSFETs with OPO and PPO is carried out. All the processes except passivation are precisely controlled to be the same. The post oxidation was carried out after the Al_2_O_3_ block layer deposition that is different from [9] in which the post oxidation was after HfO_2_ deposition. The mobility degeneration mechanism of *Coulomb* and roughness scattering is investigated. The impact of the thickness of the Al_2_O_3_ block layer on device performance is also discussed. Overall, we demonstrate that OPO is a promising passivation technique for future Ge MOSFET fabrication.

## Methods

**Fig. 1:**
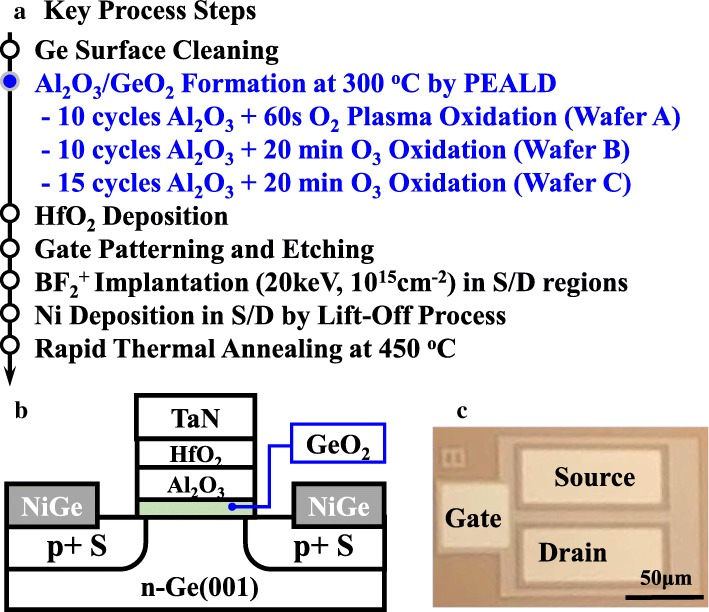
**a** Key process flow for fabricating Ge pMOSFETs with GeO_2_ surface passivation with three different passivation methods. **b** Schematic and **c** microscope images of the fabricated Ge transistor

Ge pMOSFETs were fabricated on 4-in. n-Ge (001) wafers with a resistivity of 0.14–0.183 Ω cm. Three different passivation processes were performed, and the key process steps are shown in Fig. 1a. The wafers were cleaned by diluted HF (1:50) and deionized water for several cycles to remove the native oxide and then transferred into a plasma-enhanced atomic layer deposition PEALD (Picosun R200 Advanced) chamber immediately. Then, a thin Al_2_O_3_ film (~ 1 nm) was deposited at 300 °C with trimethylaluminium (TMA) and deionized water (H_2_O) as the precursors of Al and O, respectively. Because the Al_2_O_3_/GeO_2_ layer is too thin to have a precise oxygen atom ratio, we marked these two layers as AlO_*x*_/GeO_*x*_. PPO was performed with the Litmas remote plasma source for 60 s. An ozone generator (IN USA AC series Ozone generators) with the input oxygen flow of 750 sccm was used for the OPO treatment in 50% O_3_/O_2_ ambient. Without breaking the vacuum, 60-cycle HfO_2_ was then deposited on the top of AlO_*x*_/GeO_*x*_ after PPO or OPO treatment at 300 °C using tetrakis dimethyl amino hafnium (TDMAHf) and H_2_O as the precursors of Hf and O, respectively. A 100-nm TaN was then deposited by reactive sputtering as gate metal. After gate patterning and etching, self-aligned BF^2+^ implantation into source/drain(S/D) regions with an energy of 20 keV and a dose of 1 × 10^15^ cm^− 2^ was carried out. A 20-nm Ni S/D metal was deposited and patterned by a lift-off process. Finally, rapid thermal annealing at 450 °C for 30 s for dopant activation and S/D ohmic contact was followed. The schematic and microscopy images of the fabricated Ge pMOSFETs are shown in Fig. 1b and c, respectively.

**Fig. 2:**
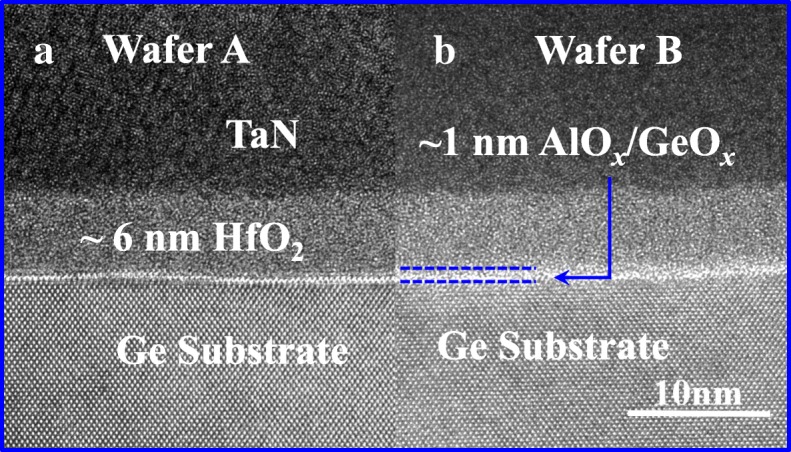
Cross-sectional TEM images of the high-k/metal gate stack with a AlO_*x*_/GeO_*x*_ interfacial layer (IL) fabricated by **a** OPO and **b** PPO on a n-Ge (001) channel

The cross section of TaN/HfO_2_/AlO_*x*_/GeO_*x*_/Ge gate stack was depicted using a transmission electron microscope (TEM) to compare the impact of oxygen plasma or ozone on GeO_*x*_ formation. Figure 2a and b show the cross-sectional TEM images of TaN/HfO_2_/AlO_*x*_/GeO_*x*_/Ge gate stack with PPO and OPO, respectively. The thickness of the amorphous HfO_2_ layer in both devices is 6 nm. Wafer A with 60s PPO treatment have a distinct AlO_*x*_/GeO_*x*_ layer between the HfO_2_ and Ge substrates. This AlO_*x*_/GeO_*x*_ layer in wafer B formed by 20 min OPO has an untidy margin. The thickness of the GeO_*x*_ layer is in accordance with the data in [10].

## Results and Discussion

**Fig. 3:**
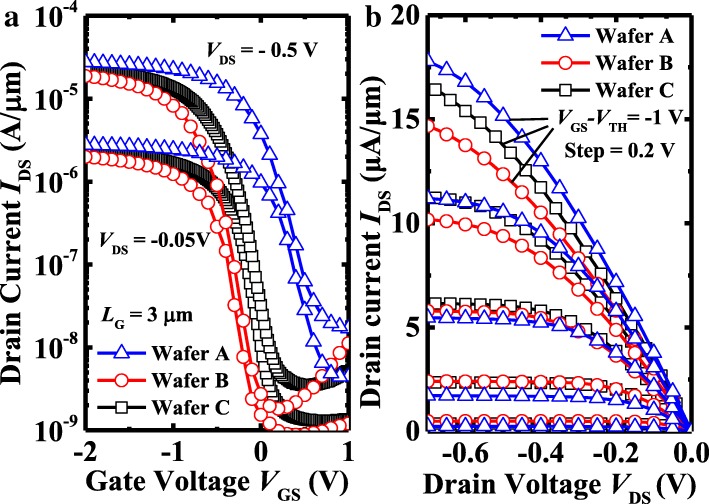
**a** *I*_DS_*–V*_GS_ and **b** *I*_DS_*–V*_DS_ characteristics of Ge pMOSFETs with a Al_2_O_3_/GeO_2_ passivation layer fabricated by PPO (wafer A) and OPO (wafers B and C)

The output and transfer characteristics coupled with high-frequency gate-to-source capacitance-voltage (CV) were measured by Keithley 4200-SCS. Figure 3 shows the comparison of transfer and output characteristic of Ge pMOSFETs with three different formation conditions of the AlO_*x*_/GeO_*x*_ passivation layer. All the devices on various wafers have a gate length (*L*_G_) of 3 μm. Devices on wafer A exhibit a higher saturated drain current (*I*_DS_) compared to the other two wafers. But wafers B and C with OPO show a much lower OFF-state current (*I*_OFF_) compared with wafer A with PPO. It is also seen that wafers B and C with OPO worked in enhancement mode and wafer A worked in depletion mode. It is inferred that, after PPO treatment, the n-Ge surface still remains to be p-type due to the high *D*_it_ value which has been discussed in [11]. Wafer C with a thicker Al_2_O_3_ block layer shows a positive *V*_TH_ shift compared with wafer B and a higher *D*_it_ than wafer B. It is observed from the output characteristics shown in Fig. 3b that, under a low gate voltage (*V*_GS_), wafer A has a lower *I*_DS_ over wafers B and C due to the less-steep subthreshold swing (SS). When the *V*_GS_ increases, *I*_DS_ of wafer A is getting higher in comparison with the other two devices. Therefore, from Fig. 3 and TEM images in Fig. 2, the diffusion of the AlO_*x*_/GeO_*x*_ layer may suppress the *I*_OFF_, thus resulting in an improvement of passivation effects.

**Fig. 4:**
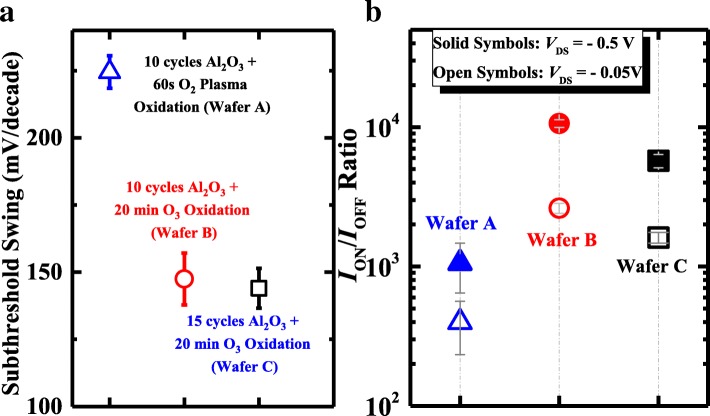
Statistical plots of **a** SS and **b** *I*_ON_*/I*_OFF_ ratio for Ge pMOSFETs with different passivation conditions

Figure 4 summarizes the statistical results of the *I*_ON_/*I*_OFF_ ratio and subthreshold swing of the devices on different wafers. Ge pMOSFETs with OPO exhibit a higher *I*_ON_/*I*_OFF_ ratio (~ 4 orders of magnitude) and remarkably improved SS in comparison with PPO device, indicating a higher quality of the dielectric/channel interface. When compared with wafer B, wafer C exhibits a higher ON-state current (*I*_ON_) but a lower *I*_ON_/*I*_OFF_ ratio.

**Fig. 5:**
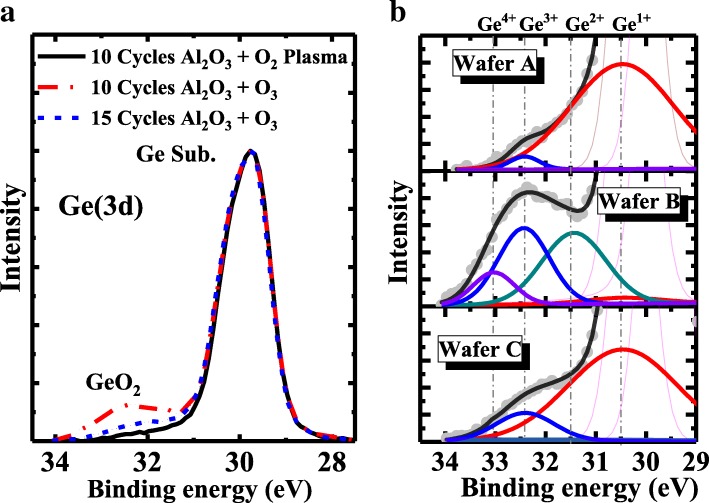
**a** Ge 3*d* XPS spectra of Al_2_O_3_/GeO_*x*_/Ge formed by different conditions. **b** Peak fittings of the Ge 3*d* XPS spectra from the GeO_2_ layer for the three samples

To further represent the interfacial layer quality of different post oxidation methods, wafers A, B, and C (dummy samples without HfO_2_ and Gate metals) were tested by X-ray photoelectron spectroscopy (XPS). XPS measurement was carried out on three post oxidation dummy samples after PPO or OPO treatment without HfO_2_ deposition and TaN sputtering. The stoichiometry of GeO_*x*_ in Al_2_O_3_/GeO/Ge samples was investigated with a monochromatic soft Al Kα (1486.6 eV) X-ray source. Ge 3*d* peaks and peak-differentiating analysis are shown in Fig. 5. The Ge 3*d*_5/2_ peak of the three samples is unified at 29.7 eV, and the chemical shifts of Ge 3*d*_3/2_, Ge^1+^, Ge^2+^, Ge^3+^, and Ge^4+^ to Ge 3*d*_5/2_ are set to 0.6, 0.8, 1.8, 2.75, and 3.4 eV, respectively [12]. In Fig. 5b, wafer A shows that after a 60s PPO, the main Ge valence in GeO_*x*_ are Ge^1+^ and Ge^3+^. A similar Ge valance state distribution is observed in wafer C, and a Ge^3+^ component is slightly increased. In Fig. 5b, wafer B shows that an OPO device with thinner (10 cycles) Al_2_O_3_ will further oxidize Ge^1+^ into Ge^2+^, Ge^3+^, and Ge^4+^, while Ge^1+^ is significantly reduced.

**Fig. 6:**
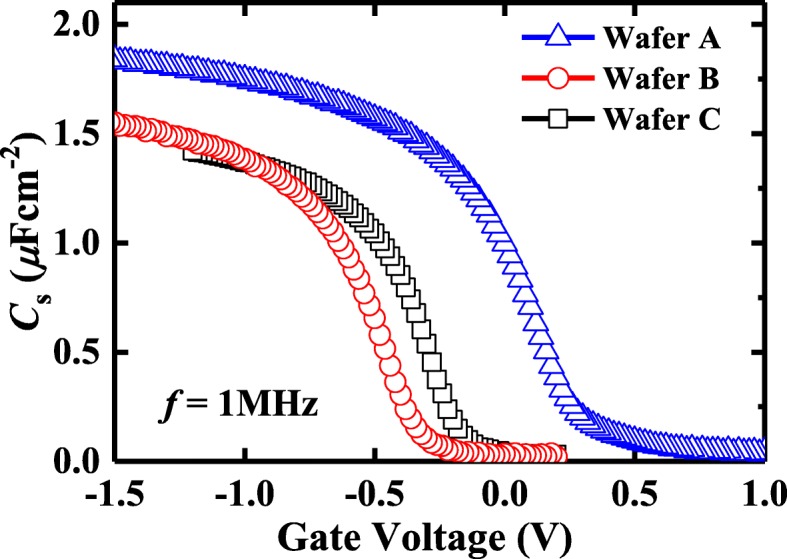
Gate-to-source capacitance versus *V*_*GS*_ characteristics of Ge pMOSFETs passivated by PPO (wafers A) and OPO (wafers B and C)

The gate-to-source CV characteristics are shown in Fig. 6. From the 1-MHz CV curve, the *D*_it_ near midgap is estimated by *Terman’s* method [13], and an equivalent oxide thickness (EOT) value is also evaluated as listed in Table 1. Twenty-minute OPO (wafers B and C) results in a higher EOT as compared with PPO (wafer A). Wafer C exhibits a higher EOT over that of wafer B, due to the thicker Al_2_O_3_ as a blocking layer. It has been reported that the thickness of GeO_*x*_ on a bare Ge surface in O_3_ ambient reaches saturation in a few minutes and the saturation thickness is dominated by temperature instead of oxidation time [10]. So in this paper, the thickness of GeO_*x*_ by ozone post oxidation is saturated after a few minutes and the left oxidation time is only for annealing.

**Table 1:** Calculated properties of Ge pMOSFETs in three passivation conditions

	*D*_it_(10^12^ cm^–2^ eV^−1^)	EOT(nm)	*R*_SD_(Ω)	*R*_CH_(Ω/μm)
Wafer A	9.07	1.83	117	32.7
Wafer B	7.91	2.11	162	46.7
Wafer C	7.46	2.13	46.7	40.1

**Fig. 7:**
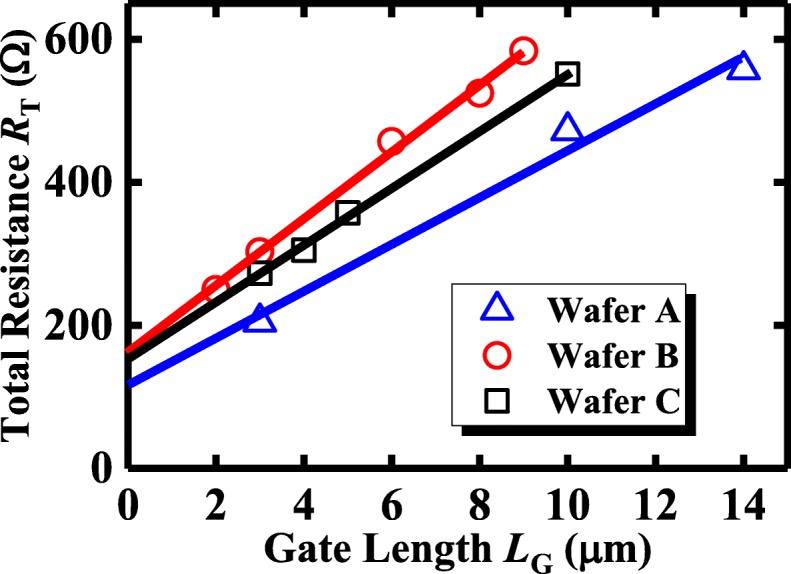
Total resistance (*R*_T_) versus gate length (*L*_G_) of Ge pMOSFETs

Figure 7 summarizes the total resistance (*R*_T_) versus *L*_G_ of each device in this work. Here, *R*_T_ is defined as *V*_DS_/*I*_DS_ at *V*_DS_ = 0.05 V and *V*_GS_ − *V*_TH_ = 1 V. The source/drain (S/D) series resistance (*R*_SD_) and channel resistance (*R*_CH_) can be extracted from the intercept and slope of the linear fitting of *R*_T_–*L*_G_ lines as shown in Fig. 7. The extracted *R*_SD_ and *R*_CH_ results are summarized in Table 1. Figure 7 shows that the Ge pMOSFETs with PPO exhibit a lower *R*_SD_ and *R*_CH_ which is consistent with the capacitance results shown in Fig. 6.

**Fig. 8:**
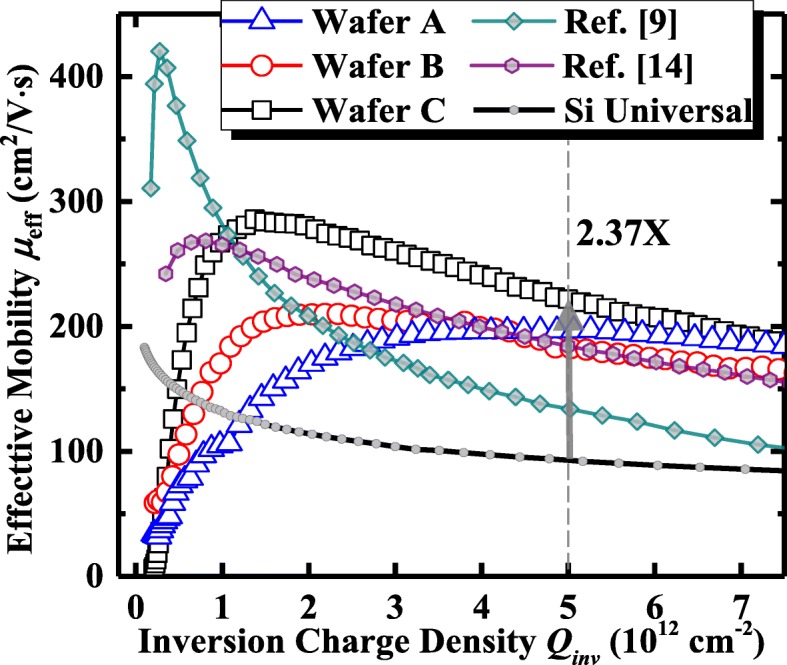
*μ*_eff_ versus *Q*_inv_ of Ge pMOSFETs with different passivation conditions. Ge transistors with 15 cycles Al_2_O_3_ + 20 min O_3_ oxidation (wafer C) exhibit a peak *μ*_eff_ of 283 cm^2^/Vs. The impact of S/D resistance on *μ*_eff_ extraction was removed by the total resistance slope-based effective channel mobility extraction method [16]

Effective hole mobility *μ*_eff_ was extracted based on a total resistance slope-based approach. In Fig. 8, we compare the *μ*_eff_ of our Ge pMOSFETs with PPO and OPO treatment with those of other reported Ge pMOSFETs [9, 14]. *Q*_inv_ is inversion charge density in the device channel. Ge pMOSFETs with OPO exhibit a higher peak *μ*_eff_ compared to the devices with PPO. Wafer C with a thicker Al_2_O_3_ block layer has a higher peak hole mobility of 283 cm^2^/V s in comparison with wafer B with the thinner Al_2_O_3_. Wafer A with PPO exhibits a lower high-field hole *μ*_eff_ with the devices with OPO, which is attributed to the lower roughness scattering. But, at low field, transistors on wafer A with PPO achieve a lower *μ*_eff_ than the OPO devices due to the higher coulomb scattering [15]. Only a few works about Ge pMOSFETs fabricated by ozone passivation have been reported. Here, a comparison of the key device performance between our devices and the reported Ge pMOSFETs treated with OPO [9, 14] are carried out, and the results are shown in Table 2. It is concluded that wafer C in this work achieves the high-field *μ*_eff_ enhancement and higher *I*_ON_/*I*_OFF_ as compared with the reported device treated with OPO. Besides, at a *Q*_inv_ of 5 × 10^12^ cm^− 2^, wafer C demonstrates a 2.37× higher *μ*_eff_ in comparison with the Si universal mobility. The *I*_ON_ of wafer C is slightly lower than that in Ref. [9] which is due to the larger EOT.

**Table 2:** Key device performance of Ge pMOSFETs in this work vs. other published results with OPO

	W/L (μm)	EOT (nm)	SS (mV/dec.)	*I*_ON_ @ *V*_DS_ = − 0.5 V *V*_GS_ − *V*_TH_ = − 0.8 V (μA/μm)	*I*_ON_/*I*_OFF_ @ *V*_DS_ = − 0.5 V	*μ*_eff_ @ peak (cm^2^/V × S)	*μ*_eff_ @ *Q*_inv_ = 5 × 10^12^ cm^−2^ (cm^2^/V × S)
Ref. [9]	−−/5	0.6	85	15.8	~ 0.9 × 10^3^	417	134
Ref. [14]	400/24	4.0	142	1.73	~ 2.3 × 10^3^	268	184
This work wafer C	100/3	2.1	144	11.2	~ 4.8 × 10^3^	283	222

## Conclusions

Ge pMOSFETs are realized with GeO_*x*_ passivation, which is formed by OPO or PPO treatment of Al_2_O_3_/n-Ge in PEALD. The OPO devices exhibit the better transfer and output characteristics, the higher *I*_ON_/*I*_OFF_ ratio, the improved subthreshold swing, and the higher peak *μ*_eff_ compared to the PPO devices. For the 15-cycle OPO process, a thicker Al_2_O_3_ layer leads to a higher EOT value and an improved *μ*_eff_ in devices compared to the 10-cycle case. All the results in this work indicate that the OPO is an effective passivation way to achieve a high-quality Ge/dielectric interface and thus can be a promising candidate passivation technique for future Ge MOSFET fabrication.

## Abbreviations

Al_2_O_3_: Aluminum oxide
ALD: Atomic layer deposition
BF_2_^+^: Boron fluoride ion
EOT: Equivalent oxide thickness
Ge: Germanium
GeO_*x*_: Germanium oxide
HF: Hydrofluoric acid
HfO_2_: Hafnium dioxide
TEM: Transmission electron microscope
MOSFETs: Metal-oxide-semiconductor field-effect transistors
OPO: Ozone post oxidation
PPO: Plasma post oxidation
*Q*_inv_: Inversion charge density
SS: Subthreshold swing
XPS: X-ray photoelectron spectroscopy
*μ*_eff_: Effective hole mobility
